# Distance Electronic Learning Strategy in Medical Teaching During the COVID-19 Pandemic: Cross-Sectional Survey Study

**DOI:** 10.2196/42354

**Published:** 2023-12-05

**Authors:** Oqba Alkuran, Lama Al-Mehaisen, Ismaiel Abu Mahfouz, Lena Al-Kuran, Fida Asali, Almu’atasim Khamees, Tariq AL-Shatanawi, Hatim Jaber

**Affiliations:** 1 Medical School The University of Jordan Amman Jordan; 2 Medical School Al-Balqa Applied University Amman Jordan; 3 Medical School Hashimite University Zarqa Jordan; 4 Medical Department King Hussein Cancer Center Amman Jordan

**Keywords:** COVID-19, distant electronic learning, medical, medicine, school, medical school, medical education, clinical skill, teaching hospital, questionnaire, distance learning, distance education, web-based education, web-based learning, medical student

## Abstract

**Background:**

Teaching hospitals have been regarded as the primary settings where doctors teach and practice high-quality medicine, as well as where medical students learn the profession and acquire their initial clinical skills. A percentage of instruction is now done over the internet or via electronic techniques. The present COVID-19 epidemic has pushed distance electronic learning (DEL) to the forefront of education at all levels, including medical institutions.

**Objective:**

This study aimed to observe how late-stage medical students felt about DEL, which was put in place during the recent COVID-19 shutdown in Jordan.

**Methods:**

We conducted a prospective, cross-sectional, web-based, questionnaire-based research study during the COVID-19 pandemic lockdown between March 15 and May 1, 2020. During this period, all medical schools in Jordan shifted to DEL.

**Results:**

A total of 380 students responded to a request to fill out the questionnaire, of which 256 completed the questionnaire. The data analysis showed that 43.6% (n=112) of respondents had no DEL experience, and 53.1% (n=136)of respondents perceived the DEL method as user-friendly. On the other hand, 64.1% (n=164) of students strongly believed that DEL cannot substitute traditional clinical teaching. There was a significant positive correlation between the perception of user-friendliness and the clarity of the images and texts used. Moreover, there was a strong positive correlation between the perception of sound audibility and confidence in applying knowledge gained through DEL to clinical practice.

**Conclusions:**

DEL is a necessary and important tool in modern medical education, but it should be used as an auxiliary approach in the clinical setting since it cannot replace conventional personal instruction.

## Introduction

Residents and medical students must be educated and trained in a teaching or university hospital, where they will have direct contact with actual patients and be exposed to real-life scenarios [1]. This concept faces ever-increasing challenges as new medical schools emerge, more students enroll, and medical specialties become more complex and require interdisciplinary teaching [2]. As a result, remote or distance electronic learning (DEL) has long been a topic of debate, and some medical schools have already made measures to construct certain electronic and virtual reality learning modules [3]. Moreover, platforms and tools have already been designed to address medical problems and requirements [4], such as videos, mobile monitoring apps [5], simulation labs [6], and wearable devices [7].

The emergence of the COVID-19 pandemic has placed an immense burden on the health care system in Jordan, resulting in an unprecedented level of stress. As a consequence, this has presented unique and complex challenges within the learning environment, both in terms of practicality and logistics. These challenges have the potential to leave a significant and enduring impact on medical education [8,9]. Web-based education holds considerable promise in terms of expanding access to education and improving efficiency; however, it does not necessarily surpass the effectiveness of traditional classroom-based approaches. The effectiveness of web-based education largely hinges on the successful integration of instructional designs that align with sound learning principles [10]. While distance education is easily implemented in theoretical courses and has been done for decades through specialized web-based platforms (eg, Coursera, EdX, and others), it is more difficult in areas that require hands-on experience. Medicine is one of these areas, and the pandemic has had a significant influence on both students and staff at medical schools, particularly in the later stages [11].

The implementation of DEL necessitates the presence of adequate digital infrastructure within each country to support and sustain these activities. This includes the necessary technological resources and systems required to facilitate effective remote learning. However, the global outbreak of COVID-19 has accelerated the widespread adoption of digital technologies in public sectors, presenting a significant challenge for governments worldwide, in addition to the health crisis [12]. Not all countries were equally equipped and prepared to address this digital transformation, exposing the varying pace of digital evolution. Societies have been compelled to adapt to this digital revolution with varying degrees of readiness. Therefore, the implications of DEL extend beyond education and encompass broader societal and ethical considerations that humanity must urgently confront and resolve, particularly given the ongoing pandemic.

This study examined the perceptions of senior medical students regarding DEL implemented during Jordan’s COVID-19 shutdown. The primary objective was to extract valuable insights that can contribute to the improvement and integration of DEL into conventional medical education, ultimately transforming it into an effective teaching tool. By exploring the experiences and feedback of medical students during this unprecedented period of remote learning, this study aimed to shed light on the strengths, weaknesses, and potential enhancements of the DEL approach in medical education.

## Methods

### Study Design

This cross-sectional study was conducted during the COVID-19 pandemic lockdown in Jordan, which lasted from March 15 to May 7, 2020. The study aimed to assess the perceptions of medical students regarding the implementation of DEL during this period.

### Participants

The target sample included fourth-, fifth-, and sixth-year medical students from all 6 medical schools in the country, encompassing various training specialties. The total estimated number of eligible participants in the national sample was around 3500-4000 students. The sample size for the study was calculated using Raosoft software. The study population (n=700) was entered into the software, and the recommended sample size was 245. In this study, a total of 700 students were given the questionnaire. Out of these, 256 students completed the survey and provided their responses, equaling a response rate of 36.6%.

### Questionnaire Development

Content with face validity is defined as that which “simply looks relevant to the person taking the test. It evaluates the appearance of the questionnaire in terms of feasibility, readability, consistency of style and formatting, and the clarity of the language used” [13]. To gather data, we developed a comprehensive questionnaire. The questionnaire underwent a face validity assessment by 5 independent academic members from the our medical school who were not involved in the study. The questionnaire was improved based on the suggestions provided by the experts. They provided their feedback by proposing some modifications, deletions, and additions. We documented all these proposed amendments, performed them, and then adopted them to conduct the study. Additionally, feedback from 20 clinical students was incorporated into the final version. We further ensured that the questionnaire, as a tool for data collection, was delivered to the participants without errors or ambiguity. The questions included were directly collected after the responses were sent from the participants. All these approaches accommodated the validity and reliability of the instrument adopted.

The questionnaire consisted of 5 sections addressing the following different aspects of the student’s experiences with DEL:

1. Past DEL experiences: Participants were asked to rank their previous experiences with DEL on a 4-point scale (none, little, adequate, or excellent).
2. Applications used: Students were provided with a list of applications commonly used by various medical schools during the lockdown, including Microsoft Teams, Zoom, WhatsApp, Skype, Google Forms, Face Life, PowerPoint with sound effects, Moodle, Videos and YouTube, and Lark. They were asked to indicate which applications they used.
3. Perceptions of the DEL method: This domain focused on the student’s perceptions of the quality and value of the DEL method. They assessed aspects such as user-friendliness, audio-visual quality, clarity, utility, and the potential for DEL to replace live or traditional teaching or be integrated into the future curriculum. Participants responded using a 5-point Likert scale.

### Validity and Reliability

The reliability of the questionnaire was tested using Cronbach α, and the value of the stability coefficient was calculated to be .871, which was acceptable.

### Data Collection

The verified questionnaire was distributed via the formal social networking sites used by all medical students in the country (including Facebook, LinkedIn, and Instagram) 4 weeks after the implementation of DEL. The survey remained open for 8 weeks, and a reminder email was sent 2 weeks after the initial launch to encourage participation.

### Data Analysis

The data analysis involved analyzing the participants’ perception of the quality and value of the DEL applications using SPSS Statistics version 22 (IBM). The responses on the Likert scale were analyzed by calculating the percentages of agreement for each statement. Correlation analysis was performed to examine the relationships between the different variables. The statistical measure used was the Spearman correlation test (*r_s_*), and significance levels were indicated with 2-tailed *P* values. The Spearman correlation test is not limited to continuous data; it can be used to test ordinal or categorical data. Since this study comprises a categorical data set, Spearman correlation was performed.

### Ethical Considerations

This study was approved by the ethical committee of the medical school of Al-Balqa Applied University (MD/56/41/1381) and was conducted according to the Declaration of Helsinki. Informed consent was signed by each participant after a clear understanding of the study objective was provided. The Institutional Review Board ensured that the study adhered to ethical guidelines and standards, protecting participants’ rights, confidentiality, and welfare.

## Results

The demographic information of the participants is presented in Table 1. The majority of the participants were men (n=129, 50.4%) and aged between 24-26 years (n=126, 49.2%). The students were asked to rate their past learning experience with DEL on a 4-point scale (none, little, adequate, excellent). Most participants (n=114, 45.4%) ranked their DEL experience as none, and 95 (37.8%) participants selected little. Only 16 (6.4%) students had excellent learning experiences with DEL.

Table 2 shows the perceptions of the participants toward DEL. Among the 256 respondents, 150 (58.6%) participants reported that the DEL method was user-friendly. Additionally, 162 (63.3%) participants stated that the sound was audible, and 145 (56.6%) participants mentioned that the images and texts used in the DEL applications were clear and helpful. However, it is worth noting that 64 (25%) participants had a negative perception, and an additional 110 (42.9%) participants had a somewhat negative perception regarding the technical aspects. Furthermore, 113 (44.1%) participants found the entire DEL experience enjoyable and beneficial, and 116 (45.3%) participants expressed their willingness to try it in the future. On the other hand, 150 (58.6%) participants disagreed that DEL can replace traditional teaching methods. Interestingly, despite the convenience offered by DEL, only 105 (41%) participants perceived it as helpful for applying clinical knowledge to patients.

Table 3 shows the correlation coefficient test between the perception of quality and the learning experience of the participants. The findings of the correlation coefficient analysis demonstrated that the tested variables had a positive and significant correlation. The audibility of sound and clinical examinations through videos and the participants’ learning experience were moderately correlated, which showed that the better the audio and video quality, the better and more enjoyable the students’ experience and the more likely students were to express zeal and eagerness to try DEL in the future. The perception of user-friendliness was also positively associated with learning experience. The comfortability and enjoyment of using DEL were positively correlated with learning experience. Moreover, the application of clinical knowledge to patients was strongly correlated with learning experience. Students perceived encouragement in applying their acquired knowledge in clinical practice. The results were evident in the students’ satisfaction with adopting DEL in teaching for medical schools (Table 4). The responses were most significant when students were asked for their consent to adopt DEL for further implementation of electronic learning teaching applications (*P*=.006).

Furthermore, the user-friendliness of DEL also had a significant impact on acquired knowledge (*P*=.02). Moreover, the responses of students were also satisfactory when they were asked about the substitution of clinical teaching with DEL, which significantly impacted acquired knowledge (*P*=.02). The responses were insignificant when the students were asked whether the images and text used helped them acquire knowledge (*P*=.23). The visualization of images and text also did not have a significant impact on acquired knowledge (*P*=.40). Similarly, audibility of sound (*P*=.09), clinical examination through videos (*P*=.07), understanding of the topic with videos (*P*=.10), the enjoyability of the DEL experience (*P*=.06), and the application of clinical knowledge to the patient following DEL (*P*=.29) had no satisfactory influence on acquired knowledge.

**Table 1:** Demographics and distance electronic learning experiences of the participants (n=256).

Category			Participants, n (%)
**Age (years)**			
	18-20	15 (5.8)	
	21-23	115 (45)	
	24-26	126 (49.2)	
**Gender**			
	Women	127 (49.6)	
	Men	129 (50.4)	
**Learning experience**			
	None	114 (45.4)	
	Little	95 (37.8)	
	Adequate	25 (10)	
	Excellent	16 (6.4)	
**Application used**			
	Microsoft	39 (15.5)	
	WhatsApp	8 (3.2)	
	Skype	1 (.4)	
	Zoom	70 (27.9)	
	PowerPoint	3 (1.2)	
	Face Live	3 (1.2)	
	Moodle	7 (2.8)	
	Google	5 (2)	
	Lark	9 (3.6)	
	E-learning	1 (.1)	
	Videos and YouTube	28 (11.2)	
	Multiple apps	42 (16.7)	

**Table 2:** Perceptions of the quality and value of distance electronic learning (DEL) applications (n=256).

Statement	Strongly disagree, n (%)	Disagree, n (%)	Neutral, n (%)	Agree, n (%)	Strongly agree, n (%)
DEL method was user-friendly	36 (14.3)	14 (5.6)	44 (17.5)	64 (25)	86 (34.3)
The sound was audible	35 (13.9)	18 (7.2)	32 (12.7)	65 (25.9)	97 (38.6)
The images and texts used were clear	30 (12)	34 (13.5)	42 (16.7)	62 (24.7)	83 (33.1)
Images and texts used were helpful	28 (11.2)	35 (13.9)	34 (13.5)	68 (27.1)	85 (33.9)
Videos helped explain the clinical examination	93 (37.1)	17 (6.8)	43 (17.1)	44 (17.5)	52 (2.7)
Videos helped me understand the topic	58 (23.1)	26 (1.4)	50 (19.9)	58 (23.1)	56 (22.3)
DEL can substitute clinical teaching	105 (41.8)	45 (17.9)	44 (17.1)	26 (1.4)	27 (1.8)
DEL’s addition to clinical teaching would be beneficial	53 (21.1)	29 (11.6)	50 (19.9)	30 (12)	84 (33.5)
DEL was an enjoyable experience	64 (25.5)	26 (1.4)	47 (18.7)	38 (15.1)	75 (29.9)
Willing to try DEL in the future	52 (2.7)	31 (12.4)	49 (19.5)	41 (16.3)	75 (29.9)
DEL experience was beneficial	55 (21.9)	29 (11.6)	55 (21.9)	49 (19.5)	59 (23.5)
DEL experience can help in applying clinical knowledge to patients	67 (26.7)	40 (15.9)	60 (23.9)	47 (18.7)	37 (14.7)

**Table 3:** Correlation analysis (Spearman rho [rs] and 2-tailed *P* value) of the perception of the quality and value of distance electronic learning (DEL).

			Learning experience		Application used		DEL method was user-friendly		The sound was audible		The images and texts used were clear		Images and texts used were helpful		Videos helped explain the clinical examination		Videos helped me understand the topic		DEL can substitute clinical teaching		DEL’s addition to clinical teaching would be beneficial		DEL was an enjoyable experience		DEL experience was beneficial		Willing to try DEL in the future		DEL experience can help in applying clinical knowledge to patients
**Learning experience**																													
	*r*_s_	1		0.153		0.208		0.044		0.072		0.154		0.157		0.145		0.187		0.193		0.155		0.181		0.221		0.110	
	*P* value	—^a^		.02		<.001		.49		.25		.02		.01		.02		<.001		<.001		.01		<.001		<.001		.08	
**Application used**																													
	*r*_s_	0.153		1		0.065		0.036		0.112		0.149		0.083		0.063		­–0.024		–0.005		0.050		0.053		0.049		0.020	
	*P* value	.02		—		.30		.56		.08		.02		.19		.32		.70		.93		.43		.40		.44		.75	
**DEL method was user-friendly**																													
	*r*_s_	0.208		0.065		1		0.550		0.549		0.580		0.375		0.450		0.439		0.557		0.575		0.633		0.607		0.243	
	*P* value	<.001		.30		—		<.001		<.001		<.001		<.001		<.001		<.001		<.001		<.001		<.001		<.001		<.001	
**The sound was audible**																													
	*r*_s_	0.044		0.036		0.550		1		0.611		0.666		0.475		0.586		0.452		0.525		0.594		0.646		0.585		0.287	
	*P* value	.49		.56		<.001		—		<.001		<.001		<.001		<.001		<.001		<.001		<.001		<.001		<.001		<.001	
**The images and texts used were clear**																													
	*r*_s_	0.072		0.112		0.549		0.611		1		0.822		0.568		0.631		0.453		0.519		0.639		0.657		0.584		0.231	
	*P* value	.25		.08		<.001		<.001		—		<.001		<.001		<.001		<.001		<.001		<.001		<.001		<.001		<.001	
**Images and texts used were helpful**																													
	*r*_s_	0.154		0.149		0.580		0.666		0.822		1		0.545		0.633		0.447		0.530		0.646		0.668		0.588		0.303	
	*P* value	.02		.02		<.001		<.001		<.001		—		<.001		<.001		<.001		<.001		<.001		<.001		<.001		<.001	
**Videos helped explain the clinical examination**																													
	*r*_s_	0.157		0.083		0.375		0.475		0.568		0.545		1		0.730		0.377		0.453		0.558		0.551		0.485		0.244	
	*P* value	.01		.19		<.001		<.001		<.001		<.001		—		<.001		<.001		<.001		<.001		<.001		<.001		<.001	
**Videos helped me understand the topic**																													
	*r*_s_	0.145		0.063		0.450		0.586		0.631		0.633		0.730		1		0.472		0.535		0.642		0.663		0.599		0.249	
	*P* value	.02		.32		<.001		<.001		<.001		<.001		<.001		—		<.001		<.001		<.001		<.001		<.001		<.001	
**DEL can substitute clinical teaching**																													
	*r*_s_	0.187		–0.024		0.439		0.452		0.453		0.447		0.377		0.472		1		0.688		0.634		0.701		0.674		0.324	
	*P* value	<.001		.70		<.001		<.001		<.001		<.001		<.001		<.001		—		<.001		<.001		<.001		<.001		<.001	
**DEL’s addition to clinical teaching would be beneficial**																													
	*r*_s_^a^	0.193		–0.005		0.557		0.525		0.519		0.530		0.453		0.535		0.688		1		0.696		0.755		0.774		0.274	
	*P* value	<.001		.93		<.001		<.001		<.001		<.001		<.001		<.001		<.001		—		<.001		<.001		<.001		<.001	
**DEL was an enjoyable experience**																													
	*r*_s_	0.155		0.050		0.575		0.594		0.639		0.646		0.558		0.642		0.634		0.696		1		0.808		0.811		0.315	
	*P* value	.01		.43		<.001		<.001		<.001		<.001		<.001		<.001		<.001		<.001		—		<.001		<.001		<.001	
**DEL experience was beneficial**																													
	*r*_s_	0.181		0.053		0.633		0.646		0.657		0.668		0.551		0.663		0.701		0.755		0.808		1		0.820		0.291	
	*P* value	<.001		.40		<.001		<.001		<.001		<.001		<.001		<.001		<.001		<.001		<.001		—		<.001		<.001	
**Willing to try DEL in the future**																													
	*r*_s_	0.221		0.049		0.607		0.585		0.584		0.588		0.485		0.599		0.674		0.774		0.811		0.820		1		0.282	
	*P* value	<.001		.44		<.001		<.001		<.001		<.001		<.001		<.001		<.001		<.001		<.001		<.001		—		<.001	
**DEL experience can help in applying clinical knowledge to patients**																													
	*r*_s_	0.110		0.020		0.243		0.287		0.231		0.303		0.244		0.249		0.324		0.274		0.315		0.291		0.282		1	
	*P* value	.08		.75		<.001		<.001		<.001		<.001		<.001		<.001		<.001		<.001		<.001		<.001		<.001		—	

^a^Not applicable.

**Table 4:** Significance of the response rate related to distance electronic learning (DEL).

Statement	Mean square error	*F* (df)	*P* value
DEL method was user-friendly	6.413	2.937 (255)	.02
The sound was audible	4.281	1.990 (255)	.10
The images and texts used were clear	1.944	1.020 (255)	.40
Images and texts used were helpful	2.707	1.415 (255)	.23
Videos helped explain the clinical examination	5.610	2.220 (255)	.07
Videos helped me understand the topic	4.351	1.953 (255)	.10
DEL can substitute clinical teaching	5.627	2.898 (255)	.02
DEL’s addition to clinical teaching would be beneficial	6.889	2.755 (255)	.03
DEL was an enjoyable experience	5.552	2.265 (255)	.06
DEL experience was beneficial	6.652	3.017 (255)	.02
Willing to try DEL in the future	9.044	3.959 (255)	.004
DEL experience can help in applying clinical knowledge to patients	2.456	1.254 (255)	.29

## Discussion

### Principal Findings

The main findings of this study regarding the influence of DEL on senior medical students during the COVID-19 shutdown in Jordan are as follows. First, a large number of participants (n=114, 45.4%) had no prior experience with this type of learning and were compelled to pursue it due to the circumstances. Despite being relatively unfamiliar with remote learning, the low number of unfavorable responses suggested that the students were willing to try this new teaching style or at least acknowledge its necessity and usefulness. It is worth noting that Jordanians have a high level of computer literacy, with a significant portion of the population owning smartphones and using social media platforms.

The results demonstrated that there was a significant and positive correlation between the perception of quality and the learning experiences of students. The audio and video quality was moderately associated with learning experience, while the application of clinical knowledge obtained through DEL to patients was strongly correlated with learning experience. Furthermore, enjoyable and comfortable experiences of DEL were strongly associated, and students were eager to try DEL in the future.

### Comparison to Prior Work

Given the mainly unfamiliar audience, the low number of unfavorable replies suggests that students were eager to attempt the new teaching style or at the very least appreciated its necessity and use. Furthermore, Jordanians are computer savvy, with 38% of the population owning a smartphone and 84% using social media sites, such as Facebook and Twitter [14]. Still, the participants in our study almost exclusively used Microsoft Teams and Zoom despite the wide variety of options.

On the subject of usefulness, the participants were rather reluctant, with only 42% rating the usefulness of DEL as positive or rather positive, while 36% rated it as negative or rather negative. They seemed fearful of incorporating this methodology into their “normal” curriculum. This was also depicted in the negative stance of 64% of students to the possibility of substituting classic teaching with DEL. The negative and positive responses were equally divided regarding both the enjoyment of the experience and the possibility of trying it in the future (roughly 40% of each group for each question).

The transformation of education from formal person-to-person classroom interactions to web-based informal meetings has greatly impacted medical education worldwide. Students are not accustomed to this type of learning, so it is not surprising that only 30% maintained a “regular” studying schedule, while 40% admitted that their schedule was upsetting. Studying is an integral part of medical education, with top students studying 6-8 hours per day in the preclinical stage [15]. Nevertheless, it is essential to diversify the concept of learning and incorporate the ever-changing conditions, either in the form of advancement or in the form of disruption [16].

Aside from the academic value of face-to-face learning, returning to traditional, on-site teaching provides much-needed social interaction for youths. When we analyzed student-patient relations, 60% missed direct in-person interactions and 77% missed their hospital-based clinical rounds. In a similar study in Singapore, two-thirds of medical students of all stages favored returning to the classrooms rather than continuing remote learning [17]. Another study of Swiss students before and after the COVID-19 lockdown showed increased levels of stress, anxiety, loneliness, and depression, with female students experiencing the symptoms more intensely [18].

Overall, the first attempt at medical teaching through DEL has received a positive note, both on a technical level as well as on the content. Luckily, digitization in Jordan is extensive and the infrastructure is in place to ensure technical efficiency throughout the country [19]. In terms of content, the migration of the course to a digital form did not reduce its quality and educational value. However, in terms of clinical value, the results were worrisome since only one-third of the students felt they could apply their knowledge to actual patients [20].

Apart from the theoretical knowledge medical students receive, they also are taught, through the example of their mentors, how to interact with patients. Clinical rounds help them familiarize themselves with medicine-in-practice in everyday situations. Additionally, peer discussions with fellow students are an effective method of deep learning in 81% of male and 89% of female students [15]. Clinical practice is based on observation, examination, investigation, and critical assessment to identify proper differential diagnoses, diagnose, treat, and sometimes operate on patients. All these skills can only be obtained by hands-on application and live peer interaction. This is the reason why only 39% of students were comfortable with the knowledge gained from DEL. Maybe more digital interaction should be implemented as a mandatory course or training to make the students more familiar with modern technologies and prepare them for the future when digital health will become mainstream.

### Strengths and Limitations

This study highlights the technical capability of Jordan to support digital medical training and recognizes the value and significant contribution of DEL in continuing medical education during the COVID-19 pandemic. However, the findings indicate that the students in this study showed reluctance and fear toward digitization, suggesting the need for further efforts to familiarize them with this educational approach. While DEL cannot fully replace traditional clinical teaching, it should be integrated as an integral part of medical education, serving as a complementary tool in the clinical setting. This study conducted a comprehensive data analysis, exploring multiple dimensions of DEL, such as prior experience, user-friendliness, and the potential for substitution of traditional clinical teaching. This thorough analysis provides a nuanced understanding of students’ attitudes and perceptions regarding DEL. Furthermore, the study’s focus on a timely and relevant topic, namely, the impact of DEL during the COVID-19 pandemic, highlights its practical implications for medical education. The findings shed light on the role of DEL as a necessary and valuable tool in modern medical education, while also emphasizing the need to address students’ concerns and integrate DEL as an auxiliary approach in the clinical setting. By offering valuable insights and recommendations, this study has the potential to inform and guide educational strategies in similar contexts.

This study has several limitations that should be acknowledged. First, the research was conducted during the COVID-19 pandemic lockdown, which created an unprecedented and unique educational context. The findings may be influenced by the specific circumstances of the pandemic and the abrupt shift to DEL, limiting their generalizability to other periods or nonpandemic situations. Moreover, the study relied on a self-report questionnaire, introducing the possibility of response bias. Participants may have provided answers that they believed were expected or socially desirable, potentially affecting the accuracy and reliability of the data. Third, the study focused on senior medical students in Jordan, which may limit the generalizability of the findings to other populations or educational settings. The cultural, institutional, and educational context of Jordan may have specific influences on students’ perceptions and experiences with DEL that differ from those in other regions. Additionally, the study did not assess the long-term effects or outcomes of DEL on students’ learning and clinical skills, which could provide further insights into the effectiveness and limitations of this educational approach.

### Future Directions

DEL is rapidly infiltrating medical schools, and this fact should be considered while designing the curriculum for future physicians. Because of their lack of experience, the students in this study were hesitant and worried about shifting to digital medical education. We are confident that calculated gradual, modest encounters will be of great benefit to clinical teaching. Future longitudinal studies are needed to evaluate all aspects of digital learning in clinical teaching for medical students. Moreover, future research and initiatives should aim to address students’ concerns and optimize the implementation of DEL to enhance the overall medical education experience.

## Abbreviations

DEL: Distance Electronic Learning
